# Elucidating the Functional and Taxonomic Diversity of Soil Microbial Communities From Three Commercial Soybean Farms in South Africa

**DOI:** 10.1111/1758-2229.70360

**Published:** 2026-05-15

**Authors:** Khumbudzo Ndhlovu, Adeola Salawu‐Rotimi, Francina L. Bopape, Prudence N. Mtsweni, Olubukola Oluranti Babalola, Ahmed Idris Hassen

**Affiliations:** ^1^ Agricultural Research Council Plant Health and Protection, Private Bag x134 Pretoria South Africa; ^2^ Food Security and Safety Focus Area, Faculty of Natural and Agricultural Sciences North‐West University Mmabatho South Africa; ^3^ Inqaba Biotechnological Industries Pretoria South Africa; ^4^ Department of Life Sciences Imperial College London, Silwood Park Campus Burkhurst Road, Ascot Berkshire UK; ^5^ Department of Plant and Soil Sciences, Faculty of Science, Engineering and Agriculture University of Venda Thohoyandou South Africa

**Keywords:** *Bradyrhizobium*, DNA, functional genes, microbes, plant‐ soil‐ interaction, shotgun metagenomics, soils, soybean, taxonomic abundance

## Abstract

Prior to the introduction of the exotic inoculant strain of *Bradyrhizobium*, South African soils lacked the rhizobia that nodulate soybean. Five decades of soybean inoculation practice resulted in the establishment of the *Bradyrhizobium* population in many soybean growing fields. However, there is no record of the magnitude of this establishment and its impact on the taxonomic and functional abundance of other microbes. Here we use a shotgun metagenomics approach to elucidate the taxonomic and functional profiles of the soil microbes from selected commercial soybean farms in South Africa. Metagenomics of the total sequences revealed that Proteobacteria, Actinobacteria, Firmicutes, Acidobacteria and Bacteroitedes are the prevalent phyla which differed in their relative abundance. *Bradyrhizobium* was the predominant genus at all three locations. Predicted functions detected genes essential for nitrogen metabolism, including nitrogen fixation, which have been unveiled in this study at a higher rate in all locations investigated. This study uncovers the microbial communities associated with soybean soils in South Africa. The study also generated vital information on the establishment of *Bradyrhizobium* spp. in the soils of soybean farms, providing a clue on whether inoculation of soya beans is always necessary. The findings, however, warrant further field investigations before any recommendations are rendered.

## Graphical abstract

Prior to inoculation of soybeans, South African soils contain no Bradyhizobia that nodulate and fix nitrogen in soybeans. Soybean cultivation with prolonged inoculation resulted in the establishment and dominance of Bradyrhizobia in three soybean farms in South Africa as revealed by a microbial community study using shotgun metagenomics.

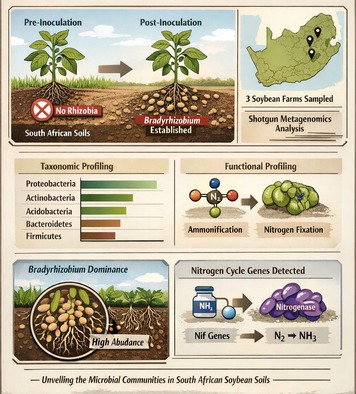

## 1 Introduction

Soil microorganisms are very diverse and play several essential roles in maintaining the health status of the soil through metabolic, nutrient cycling and organic matter breakdown processes amid a range of environmental factors (Dai et al. 2019). The interaction between plants and soil microorganisms has been thoroughly investigated for biogeochemical cycles, plant growth promotion, biocontrol, and other interactions most of which are of critical importance for sustainable soil and plant health (Wallenstein 2017; Ajiboye et al. 2022; Dlamini et al. 2022).

Plant‐microbe interactions in the rhizosphere of leguminous plants are fascinating and complex phenomena that are vital for both soil health and plant growth. Studies have shown that the legume‐rhizobia symbiotic association involves nodulation of the roots induced by the secretion of certain flavonoids by the host legumes' roots which has been investigated intensively since the discovery of the rhizobia (Shanmugam and Kingery 2018). The rhizosphere of several plant species, including legumes, is very rich in its content of microbial communities. Some of these microbes can be cultured under standard laboratory conditions and identified through molecular processes, while a greater proportion of these microbial communities contain unculturable microbes. Through the development of Next Generation Sequencing (NGS) technology, metagenomic sequencing was introduced which provides a clear insight into soil and other environmental microbes which goes far beyond the culturable microorganisms, providing a better understanding of their phenotypes, metabolic functions, and taxonomic abundance (Cai et al. 2016).

In terms of the legume‐rhizobium symbiosis of soybean ( *Glycine max* L.) in South Africa, the exotic *Bradyrhizobium diazoefficiens* WB74 strain, introduced and released as a commercial inoculant in 1998 into South African soils, is very effective in its nodulation and nitrogen fixation capacity on different soybean cultivars (Bloem 1998). Nevertheless, there is no record of the impact of its introduction into the South African soils on soil nitrogen and fertility status, as well as on the taxonomic abundance and functional diversity of the native soil microbial community. Most of the studies conducted on the legume microbe interactions in the past focused on the isolation, screening, characterization, and inoculation of rhizobia for high nitrogen fixation, plant growth, and yield. It is however worth noting that other microorganisms also play several crucial roles, including increasing nitrogen availability, soil fertility, and plant growth promotion. Many of these studies thus overlooked the overall microbial diversity associated with soybean rhizosphere in South Africa.

As there is little information on the microbial community of the soybean rhizosphere, the current study is designed to explore the soil microbial diversity, including that of the rhizobia complex at three different soybean producing fields. It is hypothesized that the microbial diversity, in particular the diversity of the rhizobia complex in the three soybean fields will be similar due to decades‐long inoculation of soybeans with an exotic strain of *Bradyrhizobium* spp. The study will be conducted on the rhizosphere soils in three commercial soybean farms in Mpumalanga and the Free State provinces of South Africa. This broader perspective can provide insights to help manage the soybean rhizosphere for enhanced soybean growth, increased productivity, and support agricultural sustainability practices.

## 2 Materials and Methods

### 2.1 Soil Sample Collection

Soil samples were collected from three different soybean growing fields in South Africa viz. Lothair (262450.9S latitude, 301926.3E longitude) and Standerton (27135152S latitude, 29448120E longitude) in the Mpumalanga province and Bothaville (−27.534286S latitude, 26.524414E longitude) in the Free State province. The soil samples were collected during the soybean planting season from November/December 2021. Approximately 1 kg of the top 6 to 8 in. of the rhizosphere soil was collected and transferred to sterile plastic bags and stored in a cooler box containing ice blocks. In total, 6 soil samples were randomly collected from different sites at each of the three soybean farms in each location. The samples were transported to the Biological Nitrogen Fixation laboratory of the Agricultural Research Council, Pretoria, South Africa. For soil physicochemical analysis, 500 g of the 6 pooled soil samples were sent for physiochemical analysis to the Agricultural Research Council‐ Institute of Soil, Climate and Water (ARC‐ISCW), Pretoria, South Africa. Another 10 g of pooled soil samples were used for shotgun metagenomics.

### 2.2 Shotgun Metagenomics

DNA extraction and sequencing were conducted following Hassen et al. (2020). Briefly, the total DNA of the soil samples was extracted from a 250 mg subsample taken randomly from the rhizosphere soil using the ZymBIOMICS DNA/RNA extraction kit. Approximately 50 ng of the DNA for each sample was used to construct the library sequence. The quality of the DNA was quantified using a Qubit dsDNA kit. DNA was subsequently randomly fragmented with a Covaris sonicator into the appropriate size range before library preparation, with the size of the DNA ranging between 300 and 500 bp for each sample. The quality of the library was assessed after the circularization of the DNA on the TruSeq Nano DNA using a high throughput preparation kit (Illumina) before sequencing on HiSeq 2500 (Illumina).

Raw FASTQ sequence files have been pre‐processed using Kraken (Wood and Salzberg 2014) and the quality of the raw data was checked using FastQC (version 0.23.1) (Andrews 2010). De novo assembly was performed with MetaSpades (Nurk et al. 2017). The quality of contigs was evaluated with Metaquast (Mikheenko et al. 2016). After preprocessing, the sequences were imported into R/RStudio version 4 (R Core Team 2024) and the (.tsv) were used for downstream analysis in R using the tidyverse, vegan, and ggplot2 packages. Taxonomic classification of the metagenomic reads was performed using Kaiju (Menzel et al. 2016), a protein‐level taxonomic classifier that assigns reads to taxa based on translated sequence similarity searches against reference microbial databases. The Kaiju output files in table separated values (.tsv) were generated for phylum, family, genus, and species levels for each site that is, Bothaville, Lothair, and Standerton.

### 2.3 Functional Profile Analysis Including Major Nitrogen Metabolism

For the functional profile analysis, contigs were annotated using Prodigal (Hyatt et al. 2010). The Evolutionary Genealogy of Genes: Non‐supervised Orthologous Groups (eggNOG) was used for functional annotation of genes generated from Prodigal. The FAMA functional profile analysis was also used to capture the genetic potential of the soil microbial communities with particular interest in nitrogen metabolism including nitrogen fixation, ammonification, nitrate assimilation, denitrification, and urease activity. The functional profile was generated using the FAMA computational tool for shotgun metagenomics data functional profiling on KBase (Arkin et al. 2018). The functional profiles between the three‐soya bean rhizosphere soil samples were compared in the EFPKG (the normalization metric for paired‐end libraries). The generated results were determined as the number of fragments per kb of effective gene length per genome equivalent (EFPKG).

### 2.4 Diversity Analysis

Alpha diversity indices (Shannon (H′), Simpson (D), and Evenness) were calculated using the *vegan* package to assess within‐sample diversity for each location and taxonomic level. Each site had a single representative Kaiju dataset, and alpha diversity indices were summarized per site. For beta diversity, the Bray–Curtis dissimilarity matrices were computed to assess compositional differences between sites. The dissimilarity matrices were visualized using NMDS ordination plots and heatmaps to show relative similarity among samples. All plots were generated in R/Rstudio, and formatted tables summarizing alpha diversity indices and Bray–Curtis distances.

## 3 Results

### 3.1 Analysis of Soil Physicochemical Properties

The soil physicochemical analysis results were indicated as a (Table S1). Soils from the soya bean growing farm, Lothair, are largely loamy, whereas those of Bothaville and Standerton were mainly sandy and clay in nature, respectively. While the soils in the Bothaville site were slightly acidic, ranging from 6.28–6.43, the soils in Lothair fall from slightly acidic to near neutral (6.31–6.94). Standerton soils have slightly acidic pH within the range of 6.43–6.54. It is also observed that Bothaville soils have a very low level of organic carbon, whereas in Standerton and Lothair, the organic carbon content was medium to high. On the other hand, the phosphorus levels were consistently high in all the soil samples. For the exchangeable cations, the level of Ca was very moderate in both Lothair and Standerton, while in Bothaville it was low. It was also observed that the level of Na was low in all three locations, while potassium and magnesium were very high. The analysis also shows the presence of a higher level of the CEC in all three locations.

### 3.2 Metagenome Sequence Analysis

The Genome assembly metrics that show the genome size, the number of contigs, the N50/L50 values and the GC content for metagenome sequences of all three locations is provided in Table S2. The total number of base pairs between the samples varied greatly in which the sequence base pairs were 54,168,517 bp for Bothaville, 44,738,967 bp for Lothair, and 53,806,625 bp for Standerton. After carrying out quality control, no sequences failed the quality control (QC) in all the samples. The predicted number of known functional gene sequences for the three locations was 2,670,017 (Bothaville), 1,371,560 (Lothair), and 2,095,656 (Standerton). The sequence showed a G + C percentage of 63.71 for Bothaville, 62.81 for Lothair, and 56.22 for Standerton.

### 3.3 Diversity of Microbial Community

**FIGURE 1:**
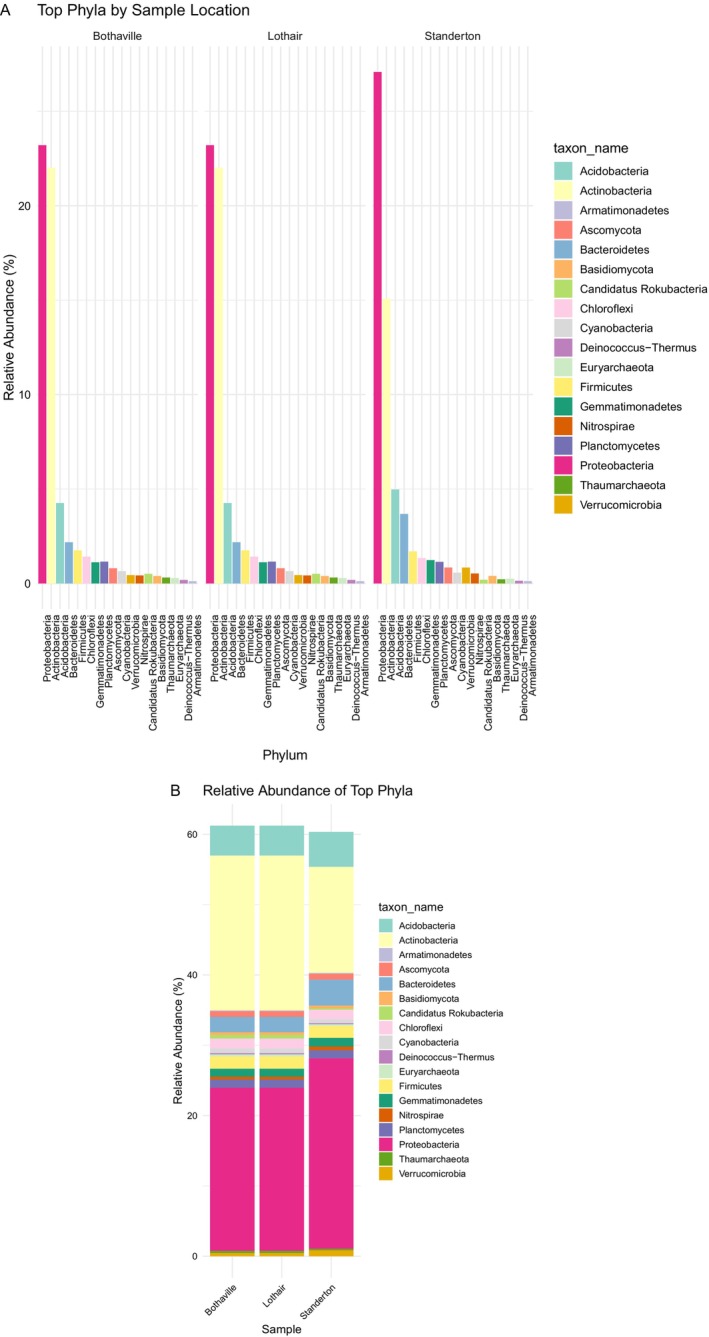
Relative abundance of top phyla with a bar chart (A) and stacked bar (B) for the three locations of soybean rhizospheres (Bothaville, Lothair, and Standerton).

**FIGURE 2:**
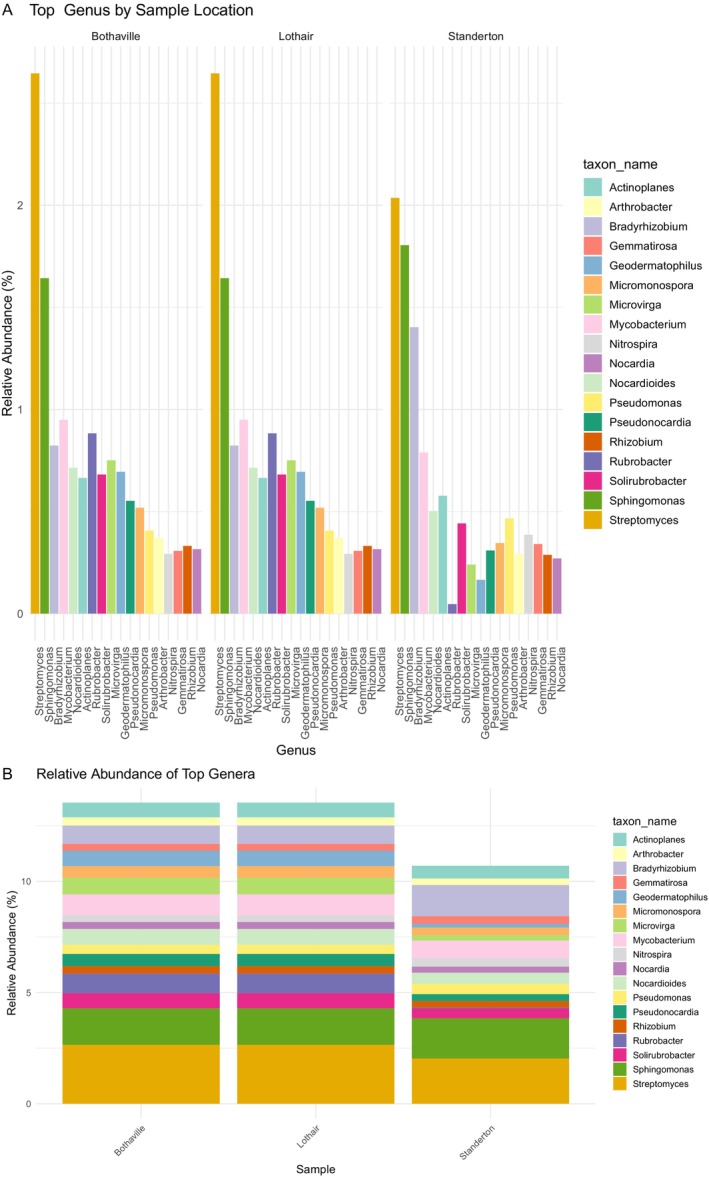
Relative abundance of top Genera of the soil microbial communities of the soybean rhizosphere in the three locations shown with a bar chart (A) and stacked bar (B).

Kaiju classification revealed that reads were successfully assigned across multiple taxonomic levels including phylum, family, genus, and species. At the phylum level, the bacterial community was dominated by members of Proteobacteria, Actinobacteria, and Acidobacteria followed by Bacteriodetes and Firmicutes at all the three locations but differed in each site by relative abundance (Figure 1A,B). At the genus level, *Streptomyces*, *Sphingomonas*, and *Bradyrhizobium* are the topmost abundant genera in Standerton, but in Lothair and Bothaville the genus *Bradyrhizobium* is outnumbered by the genera *Mycobacterium* and *Rburobacte*r, leaving *Bradyrhizobium* as the fifth most abundant genus in these locations (Figure 2A,B). Standerton soils contain the highest proportion of *Bradyrhizobium* at the genus level, almost twice the abundance recorded in Lothair and Bothaville. In addition to the genus *Bradyrhizobium*, the genus *Rhizobium* that contains the other root nodulating microsymbionts in several legumes was detected in all the three locations but at a much lower rate than the genus *Bradyrhizobium*.

**FIGURE 3:**
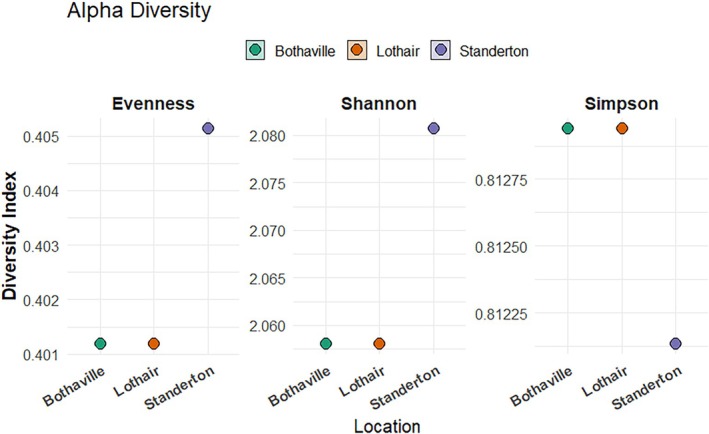
Alpha diversity indices (Shannon, Simpson, Evenness) of the soybean rhizosphere microbial communities in the three locations.

**FIGURE 4:**
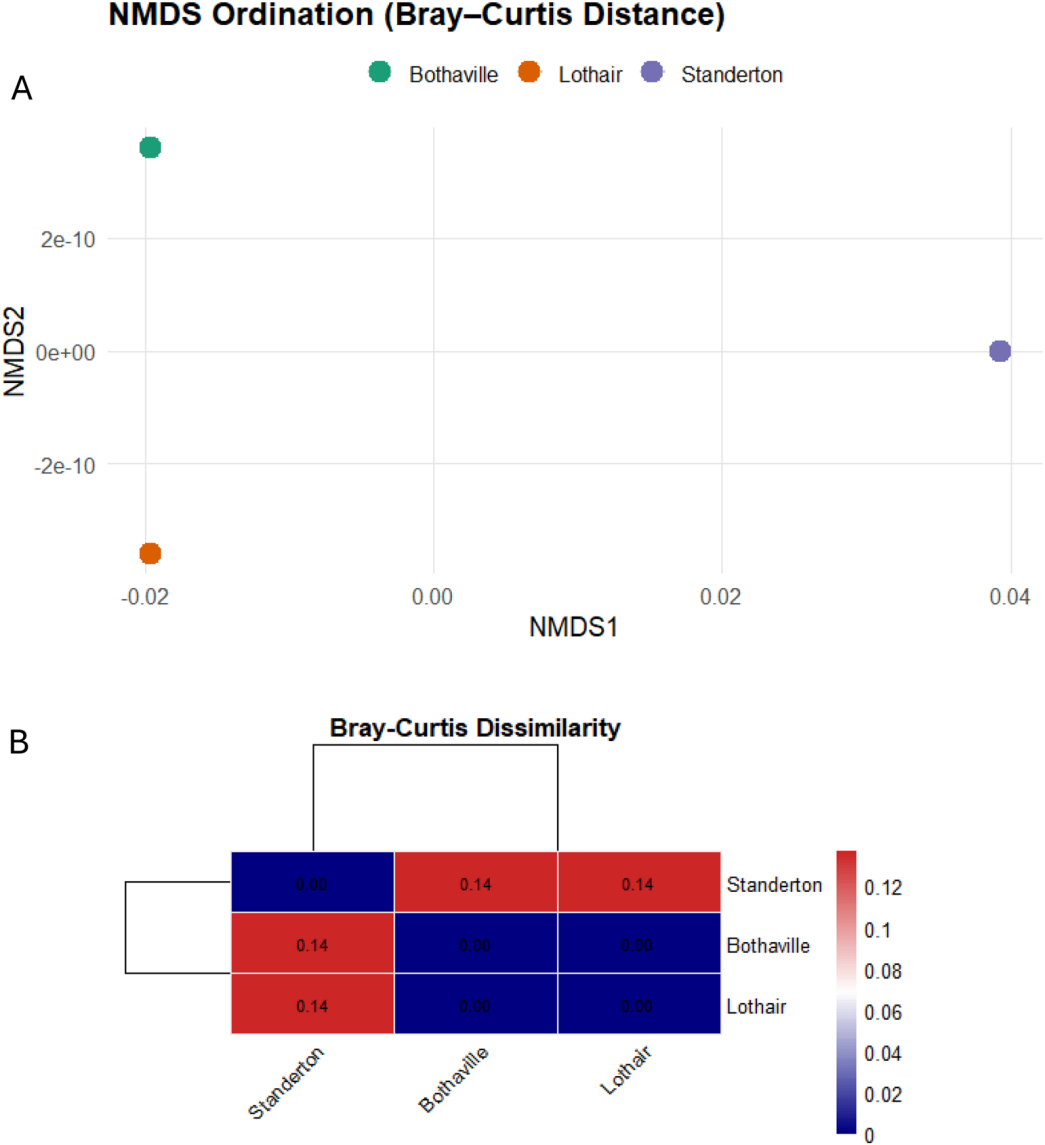
Beta diversity of the microbial communities shown by the Nonmetric multidimensional scaling ordination (NMDS) (A) and the Bray–Curtis Dissimilarity metric (B) showing how the soils in the three locations differ in species composition.

The relative abundance of the top class is dominated by Actinobacteria, followed by Alphaproteobacteria, Betaprotepbacteria, Gamaproteobacteria and Deltaproteobacteria as the topmost five classes in all the three soils of the soybean farms (Figure S4A). Bothaville soils have the highest relative abundance of Actinobacteria (28%) followed by Alphaproteobacteria (10%). whereas Lothair and Standerton soils contain the highest proportion of Betaproteobacteria, the class that contain the emerging root nodulating microsymbionts, the Burkholderia and Paraburkholderia genera. The relative abundance of the ubiquitous *Bacilli* in the soils of all three locations is much below 10%. Streptomycetaceae, Sphingomonadaceae and Bradyrhizobiaceae are the topmost abundant families at all the three soybean farms included in this study. The relative abundance of Bradyrhizobiaceae, that contains the root nodulating microsymbionts of soybeans, the *Bradyrhizobia* spp. is highest at Standerton soils but the lowest for Bothaville (Figure S4B). Despite the presence of a higher proportion of the Bradyrhizobiaceae that contains the genus *Bradyrhizobium*, the nitrogen fixing symbionts of soybeans, Lothair soils have generally the lowest relative abundance at both class and family level (Figure S4A,B). Alpha diversity metrics (Shannon, Simpson, and Evenness) were calculated to evaluate within‐site diversity (Figure 3). The results indicated that Standerton had slightly higher diversity and evenness compared with Bothaville and Lothair. Although PERMANOVA could not be computed due to a single replicate per site, Bray–Curtis dissimilarity heatmaps (Figure 4) revealed modest community differences, with Standerton showing greater dissimilarity from the other two sites.

### 3.4 Predicted Metabolic Functions Using eggNOG

The eggNOG highlights the prevalence of gene functions, functional composition and key biological processes and pathways represented in the data and identifies genes that share ancestry across species. Bar plots generated in R were used to provide a visual summary of the most frequent functional annotations from the eggNOG dataset for Bothaville, Lothair and Standerton sites. The y‐axis lists the top 10 descriptions of the genes or proteins in each dataset. These descriptions are derived from the eggNOG annotations and provide a summary of the predicted function of each gene or protein (Figures S1–S3). In Bothaville soils, transposase showed the highest count followed by the DDE superfamily endonuclease enzyme. Winged helix‐turn helix and SPTR A7NFQ2 transposase and inactivated derivatives enzyme showed a count of 14 as indicated (Figure S1). Other function annotations observed in Bothaville samples with the same count were sequence‐specific DNA binding, PFAM transposase, IS4 family protein, PFAM transposase, PFAM transposase IS116 IS110 IS902 family protein, MacB‐like periplasmic core domain, COG NOG 1460 non‐supervised Orthologs group, and binding protein‐dependent transport system inner membrane system component. In Lothair soils, transposase appeared to be high, but a little bit lower compared with Bothaville (Figures S1 and S2). Transposase activity, and reverse transcriptase belonging to the phage integrase family showed the same read count. Other functional annotations were transposase DDE domain group, transpose DDE domain, transposase and inactivated derivatives, transposase (IS116 IS110 IS902 family) pyridoxamine 5′‐phosphate oxidase and PFAM transposase. Transposase in both Lothair and Bothaville was high whereas, in the Standerton sample, it shows that it is the same as other enzymes (Figure S3). Phage portal protein shows the highest read count. Reverse transcriptase and HNH endonuclease belonging to DegT DNrJ Erycl family showed the same read count.

### 3.5 Nitrogen Metabolism Functional Genes

This study also investigated nitrogen metabolism genes including nitrogen fixation, ammonification, ammonium oxidation, denitrification, nitrate assimilatory reduction, nitrate assimilation, and urease that improve the availability of nitrogen the plants need. The comparison of selected FAMA functional profiles between the soya bean soils in the three different locations is indicated in Tables 1 and 2. Ammonium oxidation, nitrate assimilatory reduction, nitrate assimilation, and urease were found to be high in Bothaville compared with Lothair and Standerton in terms of raw sequence count (Table 1). In Lothair, raw reads sequence count for nitrogen fixation and ammonification was high (192) in contrast to Bothaville (28) and Standerton (36) with an amino acid count of 90.74% in Lothair, 90.55% in Bothaville and 88.58% in Standerton (Table 2). Denitrification was high in Standerton with a raw sequence count of 4604. Fama functional profile identified functional enzymes such as nitrogenase reductase (*nifH* with a raw sequence count of 41), Nitrogenase alpha chain (*nifD* with a raw sequence count 58), Nitrogenase beta chain (*nifK* with a raw sequence count 48), and nitrogen fixation proteins *nifB* with a raw sequence count 45 which are involved in the nitrogen cycle with the taxonomy of proteobacteria found to be higher in Lothair than in Bothaville and Standerton (Table 2). Genes like Nitrate reductase (*narG‐NxrA* linked with the taxonomy of Actinobacteria), Nitrate reductase (*nirK* with a raw sequence count of 1589) were found to be high in Standerton compared with other locations and are associated with Proteobacteria and Chloroflexota. Nitrate Reductase (*nirD* with a raw sequence count of 587) are involved in denitrification processes and found to be high in Bothaville being highly associated with *Burkholderia*, and *Solibacter* species (Table 2). Hydroxylamine dehydrogenase (*hao*) and Ammonia monooxygenase subunit A (*amoa*) are involved in nitrification which differed in location based on raw sequence reads. Other nitrogen metabolism genes were also identified.

**TABLE 1:** Functional profiles of nitrogen metabolism of soil microbial communities from the three soya bean farms based on the FAMA functional analysis.

Function category	Lothair			Bothaville			Standerton		
	Raw sequence count	EPFKG^a^	Amino acid identity (%)	Raw sequence count	EPFKG	Amino acid identity (%)	Raw sequence count	EPFKG	Amino acid identity (%)
Nitrogen fixation	192	0.27794	90.74	28	0.03410	90.55	36	0.05340	88.58
Ammonification	1158	1.87266	76.64	871	1.32238	76.97	689	1.24838	77.21
Ammonium oxidation	271	0.56714	87.1	489	0.93170	85.55	192	0.44621	90.41
Denitrification	4246	4.71150	77.89	2607	2.71569	77.41	4604	5.70185	78.48
Nitrate assimilatory reduction	3798	3.69007	77.44	4182	3.303390	77.49	3935	3.76634	85.61
Nitrate assimilation	4131	4.52261	90.74	4837	5.07001	78.84	3919	5.04758	78.14
Urease	3133	5.35362	86.41	3213	5.10504	86.62	2749	5.42779	87.07

^a^ The number of fragments per kb of effective gene length per genome‐equivalent.

**TABLE 2:** FAMA functional report on nitrogen metabolism of the soil microbial communities from the rhizosphere of the soya bean farms in Lothair, Bothaville and Standerton and representative taxonomic groups with the highest rates of the designated metabolism.

Function/Genes	Description of functions	Lothair		Standerton		Bothaville		Taxonomy with a greater number of fragments of the gene length across all soil samples
		efpkg	Raw sequence count	efpkg	Raw sequence count	efpkg	Raw sequence count	
*NifB*	Nitrogen fixation protein *NifB*	0.071	45	0.018	13	0.009	9	Proteobacteria, Beta Proteobacteria
*NifD_Anf_VnfD*	Nitrogenase alpha chain	0.073	58	0.014	10	0.009	8	Proteobacteria, Desulfuromonas, Rhizobiales
*NifH_AnfH_VnfH*	Nitrogenase reductase and maturation protein	0.074	41	0.008	4	0.008	5	α‐Proteobacteria, Rhizobiales, Bradyrhizobiaceae
*NifK_AnfK_VnfK*	Nitrogenase beta chain	0.059	48	0.012	9	0.007	6	Proteobacteria, Desulfuromonadels, Desulfuromonas
*NasA*	Assimilatory nitrate reductase large subunit	3.208	3935	3.233	3552	2.739	3683	Proteobacteria, unclassified Acidobacteria, Solibacterales, Actinobacteria, beta proteobacteria
*AmoA_PmoA*	Methane/ammonia monooxygenase subunit A	0.162	75	0.128	54	0.314	158	Proteobacteria, Archaea, Nitrospira
*AmoC_PmoC*	Methane/ammonia monooxygenase subunit C	0.256	119	0.202	81	0.409	205	Beta Proteobacteria, Nitrosomonadales, Actinobacteria, Nitrospirae, Archaea
*AmoB_AmoB*	Methane/ammonia monooxygenase subunit A	0.149	77	0.116	57	0.209	126	Archaea, alpha and beta proteobacteria, Nitrosomonadales
*NarG_NxrA*	Nitrate reductase	0.869	1526	1.011	1592	0.677	1290	Proteobacteria, unclassified Proteobacteria, Actinobacteria, Nitrospirae
*NirA*	Ferredoxin‐nitrate reductase	0.854	784	0.829	679	1.174	1148	Proteobacteria, Alphaproteobacteria, Rhizobiales, Unclassified bacteria
*NirB*	Nitrate reductase (NADH) large subunit	1.956	2471	2.162	2412	2.095	2867	Unclassified bacteria, Proteobacteria, Alphaproteobacteria
*NirD*	Nitrate reductase (NADH) small subunit	1.366	578	1.667	527	1.549	587	Proteobacteria, Burkholderiales, Actinobacteria, Solibacters
*NirK*	Nitrate reductase (NO‐ forming)	1.918	1456	2.253	1539	1.122	917	Chloroflexi, Unclasified Proteobacteria
*UreA*	Urease subunit gamma	1.536	549	1.667	520	1.471	561	Alphaproteobacterial, Actinobacteria, Unclassified Alphaproteobacteria
*UreB*	Urease subunit beta	1.617	591	1.619	513	1.523	585	Alphaproteobacterial, Rhizobiales, Actinobacteria, Unclassified Proteobacteria

Function/Genes	Description of functions	Lothair		Standerton		Bothaville		Examples of taxonomic groups with highest functions across all soya bean soil samples
		efpkg	Raw sequence count	efpkg	Raw sequence count	efpkg	Raw sequence count	
*UreC*	Urease subunit alpha	2.199	1993	2.141	1716	2.111	2067	Proteobacteria, Unclassified bacteria
*HAO*	Hydroxylamine dehydrogenase	0.057	52	0.046	37	0.061	59	Betaproteobacteria, Nitorsomonadales, Nitrospora
*NapA*	Periplasmic nitrate reductase precursor	0.533	661	0.609	672	0.353	475	Alphaproteobacteria, Rhizobiales, Unclassified Proteobacteria
*NapB*	Periplasmic nitrate reductase cytochrome	0.308	112	0.399	127	0.227	90	Betaproteobacteria, Alphaproteobacteria, Rhizobiales, unclassified Proteobacteria
*NapC*	Cytochrome c‐type protein	0.526	217	0.667	245	0.227	104	Unclassified Betaproteobacteria
*NapD*	Periplasmic nitrate reductase component	0.108	33	0.244	64	0.087	27	Proteobacteria
*NrfA*	Nitrate reductase (cytochrome c‐552)	1.121	882	0.761	531	0.775	657	Unclassified Bacteria, Proteobacteria, Deltaproteobacteria
*NrfH*	Cytochrome c nitrite reductase small subunit	0.726	265	0.477	154	0.541	211	Deltaproteobacteria, Myxococales, desulfuromonadales, Unclassified bacteria

## 4 Discussion

This report presents how the taxonomic abundance and functional diversity of rhizosphere soils from three soybean growing fields in South Africa vary based on shotgun metagenomics study. Our study reveals that soil physicochemical properties as well as cropping history influence the taxonomic abundance and functional diversity of rhizosphere microorganisms at the three sites. It is evident from other studies that physicochemical properties including texture and associated properties significantly influence the microbial diversity and composition of a given soil rhizosphere (Xia et al. 2020). For instance, when we categorically look into the microsymbionts of soybean, the highest abundance of the genus *Bradyrhizobium* was detected in the soybean rhizosphere soil of Standerton, which is characterized by high organic carbon and phosphorous (P), less available soil nitrogen (NO_3_), and a higher percentage of cation exchange capacity (CEC) including Na, K, Ca and Mg (Table S1). At all three locations, the rhizosphere soil contains sufficient phosphorous which could probably be because of established rhizobia that stimulate the host legumes to release more root exudates, which can mobilize phosphorous and increase its availability in the rhizosphere (Richardson et al. 2009; Maseko and Dakora 2013; Jaiswal et al. 2021). Compared with the relatively higher sandy textures of Bothaville and Lothair soils, the clay loam soils of Standerton are not easily flushed away and contain high levels of organic carbon which allow microorganisms to multiply at a higher rate resulting in higher microbial diversity especially during the rainy seasons (Witzgall et al. 2021). Hence, we have observed in this study that the soils in Standerton, with the intermediate texture of clay loam, are more favourable for the survival and proliferation of the root nodulating *Bradyrhizobium* which are more abundant at the genus level than in the other two locations. On the other hand, the taxonomic abundance of *Bradyrhizobium* decreased at genus level at both Bothaville and Lothair farms which have a higher proportion of sandy textured soils, which can easily dry out. These results are concurrent with the review report by Zahran (1999) that the population of rhizobia decreased in a sandy soil where the moisture level decreases and the size of the rhizobia population increased as the moisture level returns to normal.

It is very interesting, though, to observe the genus *Bradyrhizobium* as the only Alphaproteobacteria represented in the top five abundant genera at all three locations. *Bradyrhizobium*, being one of the dominant genera in all the three rhizosphere soils, helps maintain healthy soils due to its ability to fix nitrogen and to survive under abiotic stress conditions (Delmont et al. 2018; Omotayo and Babalola 2021; Omotayo et al. 2021). The dominance of *Bradyrhizobium* at all sites in this study is possibly because of the introduction of exotic rhizobia into South African soils since the early 1960s that resulted in the establishment and persistence of *Bradyrhizobium* population in major soybean farms (Figure 2A,B).

Microorganisms belonging to the phylum Proteobacteria are very common in several soils occupying the highest species richness. In this study, Proteobacteria was the most dominant phylum (Figures 1A,B) and the results concur with many other studies that reported the predominance of this phylum in the maize and soybean rhizosphere (Omotayo et al. 2022; Ajiboye et al. 2022). Although, the most abundant phylum in all the three soybean rhizospheres soils is Proteobacteria, four of the topmost abundant genera viz. *Streptomyces*, *Sphingomonas, Mycobacterium* and *Actinoplanes* belong to phylum Actinomycetota (formerly Actinobacteria). Bacteria belonging to these predominant genera often thrive in soil niches and become persistent due to their unique ecological specializations including breakdown of complex organic matter, antibiotic production, degradation of polymers and aromatic compounds (Brett et al. 2023; Jo‐Anne et al. 2024; Shuyun et al. 2025).

Actinobacteria is the second largest phylum detected in the soybean rhizosphere soils in this study. Several members of Actinobacteria are involved in improving soil health by colonizing the soil near the plant's roots, thereby creating a protective environment that promotes healthy plant growth. Some Actinobacteria groups were found in soybean nodules as endophytic partners during isolation in this study, including *Pseudonocardia*, *Rhodococcus*, *Mycobacterium*, *Microbacterium*, *Nocardia*, *Arthrobacter*, *Actinomycetes, and Archangium*. These genera can also be found in the rhizosphere of several other crops such as tomatoes, wheat, peas, and chickpeas, and many others (El‐tarabily 2008; Yu et al. 2020; AbdElgawad et al. 2020) and are known for their ability to solubilize phosphorus, produce siderophores, Aminocyclopropane carboxylic acid (ACC), and phytohormones (Lasudee et al. 2021).

The role of certain members of the Firmicutes that constitute *Bacillus* species and related genera, such as *Paenibacillus* and *Priesta* in contributing to soil fertility and act as biofertilizers for sustainable agriculture is well documented (Nikolic et al. 2025; Ichahashi et al. 2020; Omotayo et al. 2021). A recent study by Ng et al. (2025) revealed that *Bacillus* based biofertilizers enhance soil health by influencing the soil microbiome, resulting in efficient nutrient cycling and plant growth. Firmicutes have been detected frequently from all the three soybean rhizosphere soils at all locations in this study and are the fifth topmost abundant phylum. Bacteria of the genus *Gemmatirosa* within the phylum Gemmatimonadetes are not easily obtainable through culturing methods and were detected at almost similar abundance levels from all the three rhizosphere soils of soybean. Most of the bacteria in this group are not culturable and are mostly known from environmental DNA, and the first reported species *Gemmatimonas aurantiaca* strain T‐27 T was isolated from activated sludge (Zhang et al. 2003). The detection of this group of bacteria in the current study from the rhizosphere soils of soybeans is supported by a previous study (Liu et al. 2020) using metagenomics sequencing technology which confirmed their availability in the soils and their involvement in organic carbon cycling. Other groups such as Acidobacteria play a role in carbon cycling, iron cycling, and the ability to photosynthesize (Ward et al. 2009), while the processes of denitrification are performed by members of the Bacteroidetes (Chaparro et al. 2014). In addition, Chloroflexi phyla contain photosynthetic bacteria (Singh et al. 2022) that were present at higher proportions in Lothair soils.

Nitrogen is an essential element needed for plant growth, but despite its abundant presence in the atmosphere as N_2_, plants cannot use it easily because of its strong triple bond. However, leguminous plants such as soybeans can fix this atmospheric nitrogen by forming a symbiotic association with the root nodule bacteria, the rhizobia with a special preference for *Bradyrhizobium* species. It has previously been reported (Bloem 1998) that South African soils lack the specific *Bradyrhizobium* strains that can fix nitrogen with soybeans. After the introduction of exotic strains of *Bradyrhizobia* with which several soybean fields were inoculated since the 1960s, populations of *Bradyrhizobium* spp. established in several soybean‐producing fields in South Africa (Botha et al. 2004). One good observation that supports this argument is the case of the Lothair farm in this study which was last inoculated with rhizobia products 8 years ago. However, a higher microbial abundance of the nitrogen‐fixing rhizobia, mainly *Bradyrhizobium*, was observed in this location indicating the establishment and persistence of the introduced strains after several years without further inoculation. In essence, the presence of *Bradyrhizobium* enhances the nodulation efficiency in soybeans, particularly under favourable environmental conditions. It is well known that *Bradyrhizobium* species are the major symbionts of soybean and different soybean genotypes interact at varying compatibility rates with different *Bradyrhizobium* strains for nodulation and nitrogen fixation (Zheng et al. 2023). That means, not all soybean genotypes can be nodulated by a given Bradyrhizobium species but show preference or compatibility for different *Bradyrhizobium* species or strains. For instance, some cultivars nodulate efficiently with *Bradyrhizobium japonicum* , while others may form more effective symbiosis with *B. elkani*, and many of such specificity between soybean cultivars and *Bradyrhizobium species has a genetic basis* (Omari et al. 2022; Sarao et al. 2025).

While Bothaville and Lothair soils have more taxonomic abundance than Standerton soils at the genus level, such notable genera as *Rhizobium, Mesorhizobium, Arthrobacter, Pseudomonas*, and *Sphingomonas* were detected at a higher proportion. Other genera like *Devosia*, *Agrobacterium*, *Microvirga*, *Burkholderia*, *and Rhodococcus* detected in the soybean rhizosphere of at least one of the soybean farms are capable of inducing nodules in other legume hosts like Sesbania, Neptunia, Papillionoid, Mimosoid, and Lotus (Rivas et al. 2002; Cummings et al. 2009; Ampomah and Huss‐Danell 2011; Youseif et al. 2014; Dobritsa and Samadpour 2016). Another genus, which contributes to the nitrogen cycle, is *Nitrospira*, which was detected to be dominant in each location.

There is a high predominance of bacterial community than fungi at all the three soybean rhizospheres in the current study. The most predominantly detected fungal phyla were the Ascomycota followed by Basidiomycota, but with very low relative abundance of less than 2% at all the three soybean farms. Although there are no reports on the diversity of fungi in the soybean growing fields in South Africa, it is generally believed that the soybean rhizosphere secretes root exudates that are more readily metabolized by bacteria than by fungi (Cheng et al. 2025). Moreover, judging by the fact that the soybean farms included in this study have been inoculated with rhizobia for decades, the rhizobia establish a strong mutualistic association with the soybeans, which also changes the root physiology and signalling (Ren et al. 2025). These properties favour bacterial colonization over fungi, resulting in the relative abundance of the fungal communities in the soybean rhizosphere being much lower than that of the bacteria.

Alpha diversity metrics (Shannon, Simpson, and Evenness) for the three locations indicated that Standerton soil had slightly higher diversity and evenness compared with Bothaville and Lothair. The Bray–Curtis dissimilarity heatmaps also revealed modest community differences, with Standerton showing greater dissimilarity from the other two sites (Figure 2B). The differences in relative abundance of the taxa between the three locations reflect the variations in soil physicochemical characteristics that significantly influence both alpha and beta diversity of rhizosphere microbial communities. Such properties shape the ecological niches available to microbes, which in turn affect the composition and richness of the microbial community.

The current study also analysed the functional diversity of the soil samples collected from all the three soybean producing farms. As indicated in Figures S1–S3, the Bothaville soils show a very high transposase function. Live inoculants may have led to the exchange of genes with native microorganisms in the soil, causing changes in their entire genome due to the presence of transposase that influences the spread of genetic materials, thus increasing diversity regulated by the host (Mahillon and Chandler 1998; Moran and Plague 2014; Vigil‐Stenman et al. 2017). As the transposase enzyme is the most ubiquitous, it is highly controlled by the prokaryotes group. Study by Akinola et al. (2021) shows that each microbial species displays varying functions based on the acquired genes in each location.

Functional annotations using the EggNOG datasets revealed a higher distribution of transposases in the microbiomes of Lothair and Bothaville rhizosphere soils (Figures S1 and S2). This observation is crucial, particularly for the soya bean rhizosphere in Lothair farm, where inoculation with rhizobia commercial products has been disconnected for almost a decade. The occurrence of higher distribution of transposases in these soils could have resulted in the transfer of symbiotic (*nod, nif and fix*) genes from the *Bradyrhizobium* inoculant strain to the free‐living rhizobia through horizontal gene transfer (HGT) (Lemaire et al. 2015). In Standerton soils, the microbiome has a higher distribution of Phage portal proteins and HNH endonuclease both of which are also involved in facilitating horizontal gene transfer among the soil microbial communities. HNH endonuclease may assist in HGT by facilitating DNA cleavage and recombination during conjugation. Whereas phage portal proteins carry genes from one bacterium to another. Some genes spread through the soil by phage portal proteins include nitrogen‐fixation and phosphate solubilization genes. Reports in earlier studies for instance showed that, in nitrogen fixing bacteria such as rhizobia, phage portal proteins act as indispensable structural sensors that facilitate horizontal gene transfer (HGT) through transduction (Finan et al. 1984; Buchanan‐Wollaston (1979)). The introduction of a highly effective nitrogen fixing bacterium in the soil could reshape the native soil microbial community by spreading new traits through HGT including metabolic pathways (such as nitrogen fixation) and stress tolerance. This in turn can change both the composition and functional structure of the soil microbial communities with implications for soil health, plant productivity and ecosystem resilience (Hong et al. 2024; Maheshwari et al. 2017; Macedo et al. 2022).

When we look a bit deeper into the predicted nitrogen metabolism functions using the FAMA functional profile, Lothair soils have the largest raw sequence counts related to nitrogen fixation (192), in comparison to that of Bothaville (28) and Standerton (36). Likewise, the number of nitrogen fixation gene fragments per kb of effective gene length per genome equivalent (*epfkg*) is the highest for Lothair (0.27794) but several folds less for Bothaville (0.03410) and Standerton (0.05340) (Table 1). With particular emphasis on nitrogen fixation metabolism genes, Bothaville soils have the least raw sequence counts for *NifB* (Nitrogen fixation protein B), *Nif D* (Nitrogen fixation alpha chain), *Nif H* (Nitrogen reductase and maturation protein) and *NifK* (Nitrogenase beta chain) (Table 2). The analysis also revealed the abundance of *nifB*, *nifH*, *nifD* and several other nitrogen metabolism functional genes in the three locations, which once have been devoid of the rhizobia that fix atmospheric nitrogen on soybean.

Once the exotic *Bradyrhizobium* strains were introduced into soils in South Africa which never harbour rhizobia that nodulate soybeans, continuous soybean cropping gives a selective advantage allowing the rhizobia to persist for years. In doing so, even native soil bacteria acquire nitrogen fixation genes (Epstein and Tiffin 2021), resulting in an increased abundance of the *nif* genes. These results show the potential to increase nitrogen metabolism genes, and hence nitrogen fixation in the soil to improve legume yield and quality without having to apply synthetic fertilizers, but through prolonged legume cropping and inoculation with rhizobia.

## 5 Conclusion

Although South African soils were previously reported to lack the rhizobia strains that colonize and nodulate soybean roots, this study revealed for the first time that prolonged inoculation of exotic strains resulted in the establishment and persistence of *Bradyrhizobium* strains associated with soybean nodulation and nitrogen fixation. Another important finding of this study is that metagenomics sequence analysis revealed the predominance of bacterial groups belonging to Proteobacteria and Actinobacterial phyla. We have also observed that the relatively more fertile and nutrient‐rich soils of Standerton resulted in the highest abundance of *Bradyrhizobium* population and microbial diversity compared with the Lothair and Bothaville soils. It is evident that in all three locations, a higher proportion of *Bradyrhizobium* species was observed despite the variations in percentages due to several environmental factors including nutrient availability and soil properties.

The presence of a large proportion of *Bradyrhizobium* spp. capable of nodulating and fixing atmospheric nitrogen in the soybean rhizosphere is essential for sustainable soybean production, as it not only improves nitrogen fixation, growth, and yield but also reduces the dependency on external inputs such as chemical fertilizers. Through prolonged inoculation of effective nitrogen fixing strains in soils that once lacked them, a substantial establishment and persistence of the rhizobia could occur through time. The case of the Lothair farm is a perfect example in this regard, in which after prolonged inoculation, farmers discontinued the application of rhizobia inoculants since 2014. Neither did the farmers use chemical fertilizer inputs but were able to harvest as much as 3 tons per hectare due to the already established effective nitrogen fixing strains. However, given the sandy soil nature and nutrient depleted soils of Bothaville farms, it is advisable to use rhizobia inoculant products every time soybeans are planted. Generally, information on the general properties of the soil that include soil microbial abundance, functional diversity, and physicochemical characteristics is crucial in promoting sustainable agriculture, and the data generated in this study are very valuable when it comes to soybean cultivation in South Africa.

## Author Contributions

**Khumbudzo Ndhlovu:** investigation, methodology, writing – review and editing, data curation, writing – original draft. **Adeola Salawu‐Rotimi:** writing – review and editing, software, data curation, formal analysis. **Francina L. Bopape:** investigation, writing – review and editing, resources. **Prudence N. Mtsweni:** investigation, resources. **Olubukola Oluranti Babalola:** supervision, validation, writing – review and editing, investigation. **Ahmed Idris Hassen:** conceptualization, funding acquisition, validation, supervision, methodology.

## Funding

This work was supported by the National Research Foundation (NRF), South Africa, 135456.

## Conflicts of Interest

The authors declare no conflicts of interest.

## Supporting information


**Figure S1:** Distribution of the top 10 functional annotations of the metagenome in Bothaville analysed using the EggNOG Dataset.
**Figure S2:** Distribution of the top 10 functional annotations of the metagenome in Lothair analysed using the EggNOG Dataset.
**Figure S3:** Distribution of the top 10 functional annotations of the metagenome in Standerton analysed using the EggNOG Dataset.

**Figure S4:** comparison of the relative abundance of the top class (A) and family (B) of the soybean rhizosphere microbial communities between all the three locations.

**Table S1:** Physicochemical properties of the soybean rhizosphere soils from three locations.
**Table S2:** Genome Assembly Metrics for Bothaville, Lothair and Standerton.

## Data Availability

The metagenome sequence data were deposited at the NCBI database library under the Bio‐project number PRJNA1273861. The individual sample metadata are available under Biosample accession numbers [SAMN48959320—SAMN48959322].

## References

[emi470360-bib-0001] AbdElgawad, H. , W. Abuelsoud , M. M. Madany , S. Selim , G. Zinta , and A. S. Mousa . 2020. “Actinomycetes Enrich Soil Rhizosphere and Improve Seed Quality as Well as Productivity of Legumes by Boosting Nitrogen Availability and Metabolism.” Biomolecules 10: 1675. 33333896 10.3390/biom10121675 PMC7765327

[emi470360-bib-0002] Ajiboye, T. T. , A. S. Ayangbenro , and O. O. Babalola . 2022. “Functional Diversity of Microbial Communities in the Soybean ( *Glycine max* L.) Rhizosphere From Free State, South Africa.” International Journal of Molecular Sciences 23, no. 16: 9422. 36012686 10.3390/ijms23169422 PMC9409019

[emi470360-bib-0003] Akinola, S. A. , A. S. Ayangbenro , and O. O. Babalola . 2021. “Metagenomics Insight Into the Community Structure of Maize‐Rhizosphere Bacteria as Predicted by Different Environmental Factors and Their Functioning Within Plant Proximity.” Microorganisms 9, no. 7: 1419. 10.3390/microorganisms9071419. 34209383 PMC8304108

[emi470360-bib-0004] Ampomah, O. Y. , and K. Huss‐Danell . 2011. “Genetic Diversity of Root Nodule Bacteria Nodulating *Lotus corniculatus* and *Anthyllis vulneraria* in Sweden.” Systematic and Applied Microbiology 34, no. 4: 267–275. 21497473 10.1016/j.syapm.2011.01.006

[emi470360-bib-0005] Andrews, S. “FastQC: A Quality Control Tool for High Throughput Sequence Data.” 2010. http://www.bioinformatics.babraham.ac.uk/projects/fastqc/.

[emi470360-bib-0006] Arkin, A. P. , R. W. Cottingham , C. S. Henry , et al. 2018. “KBase: The United States Department of Energy Systems Biology Knowledgebase.” Nature Biotechnology 36, no. 7: 566–569. 10.1038/nbt.4163. PMC6870991 29979655

[emi470360-bib-0072] Bloem, J. 1998. “Soybean New Inoculant Strain Available.” Farmers Weekly, 16–17. Pretoria, South Africa.

[emi470360-bib-0008] Botha, W. J. , J. B. Jaftha , J. F. Bloem , J. H. Habig , and I. J. Law . 2004. “Effect of Soil Bradyrhizobia on the Success of Soybean Inoculant Strain CB 1809.” Microbiological Research 159, no. 3: 219–231. 10.1016/j.micres.2004.04.004. 15462522

[emi470360-bib-0073] Brett, R. , H. M. A. Lane , A. H. Dicko , M. R. Fulcher , and L. L. Kinkel . 2023. “Temporal Variability in Nutrient use Among Streptomyces Suggests Dynamic Nich Partitioning.” Environmental Microbiology 25: 3527–3535. 10.1111/1462-2920.16498. 37669222

[emi470360-bib-0009] Buchanan‐Wollaston, V. 1979. “Generalized Transduction in *Rhizobium leguminosarum* .” Journal of General Microbiology 112: 135–142.

[emi470360-bib-0010] Cai, M. , D. Wilkins , J. Chen , et al. 2016. “(2016) Metagenomics Reconstruction of Key Anaerobic Digestion Pathways in Municipal Sludge and Industrial Wastewater Biogas‐Producing Systems.” Frontiers in Microbiology 7: 778. 27252693 10.3389/fmicb.2016.00778 PMC4879347

[emi470360-bib-0012] Chaparro, J. M. , D. V. Badri , and J. M. Vivanco . 2014. “Rhizosphere Microbiome Assemblage Is Affected by Plant Development.” ISME Journal 8: 790–803. 24196324 10.1038/ismej.2013.196 PMC3960538

[emi470360-bib-0013] Cheng, S.‐S. , C. A. Contador , F. Zhang , Y.‐L. Ho , and H.‐M. Lam . 2025. “Whispers Beneath the Soil: Soybean‐Microbe Communication Pathways in the Rhizosphere.” Frontiers in Plant Science 16: 1686819. 10.3389/fpls.2025.1686819. 41200480 PMC12586083

[emi470360-bib-0014] Cummings, S. P. , P. Gyaneshwar , P. Vinuesa , et al. 2009. “Nodulation of Sesbania Species by Rhizobium (Agrobacterium) Strain IRBG74 and Other Rhizobia.” Environmental Microbiology 11: 2510–2525. 19555380 10.1111/j.1462-2920.2009.01975.x PMC7163632

[emi470360-bib-0015] Dai, L. , G. Zhang , Z. Yu , H. Ding , Y. Xu , and Z. Zhang . 2019. “Effect of Drought Stress and Development Stages on Microbial Community Structure and Diversity I Peanut Rhizosphere Soil.” International Journal of Molecular Sciences 20: 2265. 31071918 10.3390/ijms20092265 PMC6540327

[emi470360-bib-0016] Delmont, T. O. , C. Quince , A. Shaiber , et al. 2018. “Nitrogen‐Fixing Populations of Planctomycetes and Proteobacteria Are Abundant in Surface Ocean Metagenomes.” Nature Microbiology 3: 804–813. 10.1038/s41564-018-0176-9 PMC6792437 29891866

[emi470360-bib-0017] Dlamini, S. P. , A. O. Akanmu , and O. O. Babalola . 2022. “Rhizospheric Microorganisms: The Gateway to a Sustainable Plant Health.” Frontiers in Sustainable Food Systems 6: 925802.

[emi470360-bib-0018] Dobritsa, A. P. , and M. Samadpour . 2016. “Transfer of Eleven *Burkholderia* Species to the Genus Paraburkholderia and Proposal of Caballeronia Gen. Nov., a New Genus to Accommodate Twelve Species of Burkholderia and Paraburkholderia.” International Journal of Systematic and Evolutionary Microbiology 66: 2836–2846. 27054671 10.1099/ijsem.0.001065

[emi470360-bib-0019] El‐tarabily, K. A. 2008. “Promotion of Tomato ( *Lycopersicon esculentum* Mill.) Plant Growth by Rhizophere Compete 1‐Aminocyclopropane‐1‐Carboxylic Acid Deaminase‐Producing Streptomycete Actinomycetes.” Plant and Soil 308: 161–174.

[emi470360-bib-0020] Epstein, B. , and P. Tiffin . 2021. “Comparative Genomics Reveals High Rates of Horizontal Transfer and Strong Purifying Selection on Rhizobial Symbiosis Genes.” Proceedings of the Royal Society B 288: 20201804. 10.1098/rspb.2020.1804. 33402066 PMC7892418

[emi470360-bib-0022] Finan, T. M. , E. Hartweig , K. LeMieux , K. Bergman , G. C. Walker , and E. R. Signer . 1984. “General Transduction in *Rhizobium meliloti* .” Journal of Bacteriology 159, no. 1: 120–124. 10.1128/jb.159.1.120-124.1984. 6330024 PMC215601

[emi470360-bib-0025] Hassen, A. I. , R. Pierneef , Z. H. Swanevelder , and F. L. Bopape . 2020. “Microbial and Functional Diversity of Cyclopia Intermedia Rhizosphere Microbiome Revealed by Analysis of Shotgun Metagenomics Sequence Data.” Data in Brief 32: 106288. https://www.ncbi.nlm.nih.gov/sra/PRJNA1273861. 32984478 10.1016/j.dib.2020.106288 PMC7494673

[emi470360-bib-0026] Hong, J. , W. Xue , and T. Wang . 2024. “Emergence of Alternative Stable States in Microbial Communities Undergoing Horizontal Gene Transfer.” eLife 13: 1–23. 10.7554/eLife.99593. PMC11875537 40029705

[emi470360-bib-0027] Hyatt, D. , G. L. Chen , P. F. Locascio , M. L. Land , F. W. Larimer , and L. J. Hauser . 2010. “Prodigal: Prokaryotic Gene Recognition and Translation Initiation Site Identification.” BMC Bioinformatics 11: 119. 10.1186/1471-2105-11-119. 20211023 PMC2848648

[emi470360-bib-0028] Ichahashi, Y. , Y. Date , A. Shino , et al. 2020. “Multi‐Omics Analysis on an Agroecosystem Reveals the Significant Role or Organic Nitrogen to Increase Agricultural Crop Yield.” PNA 117, no. 25: 14552–14560. 10.1073/pnas.1917259117 PMC7321985 32513689

[emi470360-bib-0029] Jaiswal, S. K. , M. P. Maredi , and F. D. Dakora . 2021. “Rhizosphere P Enzyme Activity, Mineral Nutrients Concentration and Microbial Community Structure are Altered by Intra‐Hole Cropping of Cowpea With Cereals.” Frontiers in Agronomy 3: 666351.

[emi470360-bib-0074] Jo‐Anne, V. , M. R. J. Croese , S. E. Lakemeier , et al. 2024. “Polyester Degredation by Soil Bacteria: Identification of Conserved Bhetase Enzymes in Streptomyces.” Communications Biology 7: 710. 10.1038/s42003-024-06414. 38867087 PMC11169514

[emi470360-bib-0031] Lasudee, K. , P. Rangseekaew , and W. Pathom‐aree . 2021. “Endophytic Actinobacteria Associated With Mycorrhizal Spores and Their Benefits to Plant Growth.” Endophytes Mineral Nutrient Management V3: 229–246.

[emi470360-bib-0032] Lemaire, B. , J. Van Cauwenberghe , S. Chimphango , et al. 2015. “Recombination and Horizontal Transfer of Nodulation and ACC Deaminase (*acdS*) Genes Within *Alpha*‐ and *Betaproteobacteria* Nodulating Legumes of the Cape Fynbos Biome.” FEMS Microbiology Ecology 91, no. 11: fiv118. 10.1093/femsec/fiv118. 26433010

[emi470360-bib-0033] Liu, R. , Z. Wang , L. Wang , et al. 2020. “Bulk and Active Sediment Prokaryotic Communities in the Mariana and Mussau Trenches.” Frontiers in Microbiology 11: 1521. 10.3389/fmicb.2020.01521. 32765444 PMC7381213

[emi470360-bib-0034] Macedo, G. , A. K. Oleeson , L. Maccario , et al. 2022. “Horizontal Gene Transfer of an IncP1 Plasmid to Soil Bacterial Community Introduced by *Escherichia coli* Through Manure Amendment in Soil Microcosms.” Environmental Science & Technology 56: 11398–11408. 35896060 10.1021/acs.est.2c02686 PMC9387108

[emi470360-bib-0035] Maheshwari, M. , H. H. Abulreesh , M. S. Khan , I. Ahmad , and J. Pitchel . 2017. “Horizontal Gene Transfer in Soil and the Rhizosphere: Impact on Ecological Fitness of Bacteria.” In Agriculturally Important Microbes for Sustainable Agriculture, edited by V. Meena , P. Mishra , J. Bisht , and A. Pattanayak . Springer. 10.1007/978-980-10-5589-8-6.

[emi470360-bib-0036] Mahillon, J. , and M. Chandler . 1998. “Insertion Sequences.” Microbiology and Molecular Biology Reviews 62: 725–774. 9729608 10.1128/mmbr.62.3.725-774.1998 PMC98933

[emi470360-bib-0037] Maseko, S. T. , and F. D. Dakora . 2013. “Rhizosphere Acid and Alkaline Phosphatase Activity as a Marker of P Nutrition in Nodulated Cyclopia and Aspalathus Species in the Cape Fynbos of South Africa.” South African Journal of Botany 89: 289–295.

[emi470360-bib-0038] Menzel, P. , K. L. Ng , and A. Krogh . 2016. “Fast and Sensitive Taxonomic Classification for Metagenomics With Kaiju.” Nature Communications 7: 11257. 10.1038/ncomms11257. PMC4833860 27071849

[emi470360-bib-0039] Mikheenko, A. , V. Saveliev , and A. Gurevich . 2016. “MetaQUAST: Evaluation of Metagenome Assemblies.” Bioinformatics 32, no. 7: 1088–1090. 10.1093/bioinformatics/btv697. 26614127

[emi470360-bib-0041] Moran, N. A. , and G. R. Plague . 2014. “Genomic Changes Following Host Restriction in Bacteria.” Current Opinion in Genetics & Development 14: 627–633. 10.1016/j.gde.2004.09.003 15531157

[emi470360-bib-0042] Ng, F. L. , T. C. Lin , E. Wang , et al. 2025. “Bacillus Based Biofertilizer Influences Soil Microbiome to Enhance Soil Health for Sustainability Agriculture.” Sustainability 17: 6293. 10.3390/su17146293.

[emi470360-bib-0043] Nikolic, M. , T. Janakiev , K. Kruscic , S. Niksevic , and I. Dimkic . 2025. “Bacillus and Related Genera in Sustainable Agriculture and Their Effectiveness for Soil Health.” Bioscience, Biotechnology, and Biochemistry 90: 258–291. 10.1093/bbb/zbaf177 41313315

[emi470360-bib-0044] Nurk, S. , D. Meleshko , A. Korobeynikov , and P. A. Pevzner . 2017. “metaSPAdes: A New Versatile Metagenomic Assembler.” Genome Research 27, no. 5: 824–834. 10.1101/gr.213959.116. 28298430 PMC5411777

[emi470360-bib-0045] Omari, R. A. , K. Yuan , K. T. Anh , et al. 2022. “Enhanced Soybean Productivity by Inoculation With Indigenous Bradyrhizobium Strains in Agroecological Conditions of Northeast Germany.” Frontiers in Plant Science 12: 707080. 10.3389/fpls.2021.707080. 35095938 PMC8790476

[emi470360-bib-0046] Omotayo, O. P. , and O. O. Babalola . 2021. “Resident Rhizosphere Microbiome's Ecological Dynamics and Conservation: Towards Achieving the Envisioned Sustainable Development Goals, A Review.” International Soil and Water Conservation Research 9: 127–142. 10.1016/j.iswcr.2020.08.002.

[emi470360-bib-0047] Omotayo, O. P. , O. N. Igiehon , and O. O. Babalola . 2021. “Metagenomic Study of the Community Structure and Functional Potentials in Maize Rhizosphere Microbiome: Elucidation of Mechanisms Behind the Improvement in Plants Under Normal and Stress Conditions.” Sustainability 13: 8079. 10.3390/su13148079.

[emi470360-bib-0048] Omotayo, O. P. , O. N. Igiehon , and O. O. Babalola . 2022. “Microbial Genes of Agricultural Importance in Maize Rhizosphere Unveiled Through Shotgun Metagenomics.” Spanish Journal of Soil Science 12: 10427. 10.3389/sjss.2022.10427.

[emi470360-bib-0070] R Core Team . 2024. “R: A Language and Environment for Statistical Computing.” R Foundation for Statistical Computing, Version 4.4.1, Vienna, Austria.

[emi470360-bib-0052] Ren, H. , H. Hong , B. Zha , et al. 2025. “Soybean Productivity Can Be Enhanced by Understanding Rhizosphere Microbiota: Evidence From Metagenomics Analysis From Diverse Agroecosystems.” Microbiome 13: 105. 10.1186/s40168-025-02104-y. 40287775 PMC12034204

[emi470360-bib-0053] Richardson, A. E. , J.‐M. Barea , A. M. McNeill , and C. Prigent . 2009. “Acquisition of Phosphorous and Nitrogen in the Rhizosphere and Plant Growth Promotion by Microorganisms.” Plant and Soil 321: 305–339.

[emi470360-bib-0054] Rivas, R. , E. Velazquez , A. Willems , et al. 2002. “A New Species of Devosia That Forms a Unique Nitrogen‐Fixing Root‐Nodule Symbiosis With the Aquatic Legume Neptune Natans (Lf). Drce.” Applied and Environmental Microbiology 68: 5217–5222. 12406707 10.1128/AEM.68.11.5217-5222.2002 PMC129907

[emi470360-bib-0055] Sarao, S. K. , V. Boothe , B. K. Das , J. L. Gonzalez‐Hernandez , and V. S. Brozel . 2025. “Bradyrhizobium and Soybean Rhizosphere: Species Level Bacterial Population Dynamics in Established Soybean Fields, Rhizosphere and Nodules.” Plant and Soil 508: 515–530.

[emi470360-bib-0058] Shanmugam, S. G. , and W. L. Kingery . 2018. “Changes in Soil Microbial Community Structure in Relation to Plant Succession and Soil Properties During 4000 Years of Pedogenesis.” European Journal of Soil Biology 88: 80–88.

[emi470360-bib-0075] Shuyun, L. , J. Liu , and P. Fang . 2025. “Biodegredation of Phanantrene by Mycobacterium sp.” TJFP1: Genetic Basis and Environmental Validation. Microorganisms 13: 1171. 10.3390/microorganisms13051171. 40431342 PMC12114333

[emi470360-bib-0059] Singh, N. , V. Singh , S. N. Rai , E. Vamanu , and M. P. Singh . 2022. “Metagenomic Analysis of Garden Soil‐Derived Microbial Consortia and Unveiling Their Metabolic Potential in Mitigating Toxic Hexavalent Chromium.” Life 12: 2094. 36556458 10.3390/life12122094 PMC9781466

[emi470360-bib-0060] Vigil‐Stenman, T. , K. Ininbergs , B. Bergman , and M. Ekman . 2017. “High Abundance and Expression of Transposases in Bacteria From the Baltic Sea.” ISME Journal 11, no. 11: 2611–2623. 10.1038/ismej.2017.114. 28731472 PMC5649170

[emi470360-bib-0061] Wallenstein, M. D. 2017. “Managing and Manipulating the Rhizosphere Microbiome for Plant Health: A Systems Approach.” Rhizosphere 3: 230–232. 10.1016/j.rhisph.2017.04.004.

[emi470360-bib-0062] Ward, N. L. , J. F. Challacombe , P. H. Janssen , et al. 2009. “Three Genomes From the Phylum Acidobacteria Provide Insight Into the Lifestyle of These Microorganisms in Soils.” Applied and Environmental Microbiology 75, no. 7: 2046–2056. 19201974 10.1128/AEM.02294-08 PMC2663196

[emi470360-bib-0063] Witzgall, K. , A. Vidal , and D. I. Schubert . 2021. “Particulate Organic Matter as a Functional Soil Component for Persistent Soil Organic Carbon.” Nature Communications 12: 4115. 10.1038/s41467-021-24192-8. PMC8257601 34226560

[emi470360-bib-0071] Wood, D. E. , and S. L. Salzberg . 2014. “Kraken: Ultrafast Metagenomic Sequence Classification Using Exact Alignments.” Genome Biology 15. 10.1186/gb-2014-15-3-r46. PMC4053813 24580807

[emi470360-bib-0064] Xia, Q. , T. Rufty , and W. Shi . 2020. “Soil Microbial Diversity and Composition: Links to Soil Texture and Associated Properties.” Soil Biology and Biochemistry 149: 107953. 10.1016/j.soilbio.2020.107953.

[emi470360-bib-0065] Youseif, S. H. , F. El‐Megeed , A. Ageez , Z. K. Mogamed , A. Shamseldin , and S. A. Saleh . 2014. “Phenotypic Characteristics and Genetic Diversity of Rhizobia Nodulating Soybean in Egyptian Soils.” European Journal of Soil Biology 60: 34–43.

[emi470360-bib-0066] Yu, Z. , C. Han , B. Yu , J. Zhao , Y. Yan , and S. Huang . 2020. “Taxonomic Characterization and Secondary Metabolite Analysis of *Streptomycetes triticiradixis* sp. Nov: A Novel Actinomycete With Antifungul Activity.” Microorganisms 8: 77. 31948045 10.3390/microorganisms8010077 PMC7023189

[emi470360-bib-0067] Zahran, H. H. 1999. “Rhizobium‐Legume Symbiosis and Nitrogen Fixation Under Severe Conditions and in an Arid Climate. Microbiol.” Microbiology and Molecular Biology Reviews 63: 968–989. 10585971 10.1128/mmbr.63.4.968-989.1999 PMC98982

[emi470360-bib-0068] Zhang, H. , Y. Sekiguchi , S. Hanada , et al. 2003. “ *Gemmatimonas aurantiaca* Gen. Nov., sp. Nov., a Gram‐Negative, Aerobic, Polyphosphate‐Accumulating Micro‐Organism, the First Cultured Representative of the New Bacterial Phylum Gemmatimonadetes Phyl. Nov.” International Journal of Systematic and Evolutionary Microbiology 53: 1155–1163. 10.1099/ijs.0.02520-0. 12892144

[emi470360-bib-0069] Zheng, Q. U. , L. I. Yue‐han , X. U. Wei‐hui , C. H. E. N. Wen‐jing , H. U. Yun‐long , and W. A. N. G. Zhi‐gang . 2023. “Different Genotypes Regulate the Microbial Community Structure in the Soybean Rhizosphere.” Journal of Intergrative Agriculture 22, no. 2: 585–597.

