# Supplementary material for: Elucidating the Functional and Taxonomic Diversity of Soil Microbial Communities From Three Commercial Soybean Farms in South Africa
Source: Environ Microbiol Rep. 2026 May 15;18(3):e70360. doi: 10.1111/1758-2229.70360 (PMC13178142; doi:10.1111/1758-2229.70360)
Supplement: Supplementary file 1 — Figure S1: Distribution of the top 10 functional annotations of the metagenome in Bothaville analysed using the EggNOG Dataset. Figure S2: Distribution of the top 10 functional annotations of the metagenome in Lothair analysed using the EggNOG Dataset. Figure S3: Distribution of the top 10 functional annotations of the metagenome in Standerton analysed using the EggNOG Dataset. [file EMI4-18-e70360-s002.pdf]

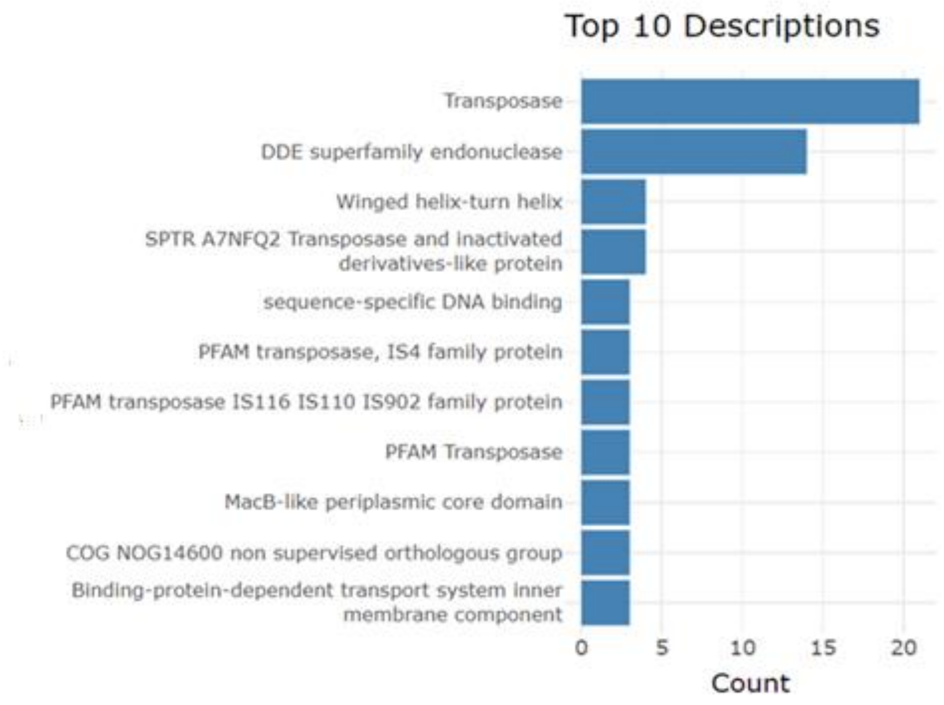

Figure S1: Distribution of the top 10 functional annotations of the metagenome in Bothaville analysed using the EggNOG Dataset.

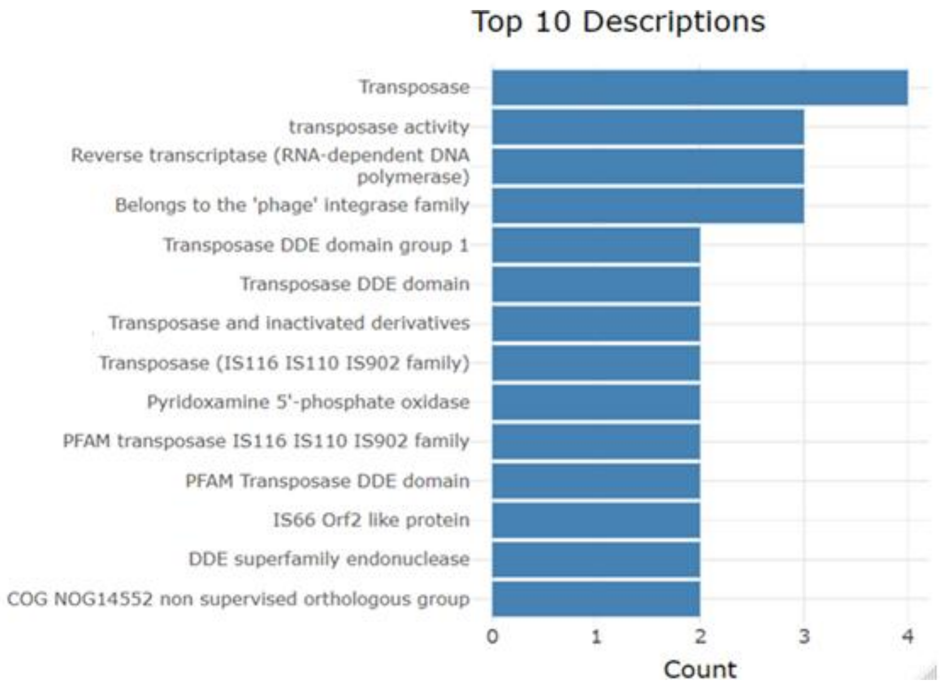

Figure S2: Distribution of the top 10 functional annotations of the metagenome in Lothair analysed using the EggNOG Dataset.

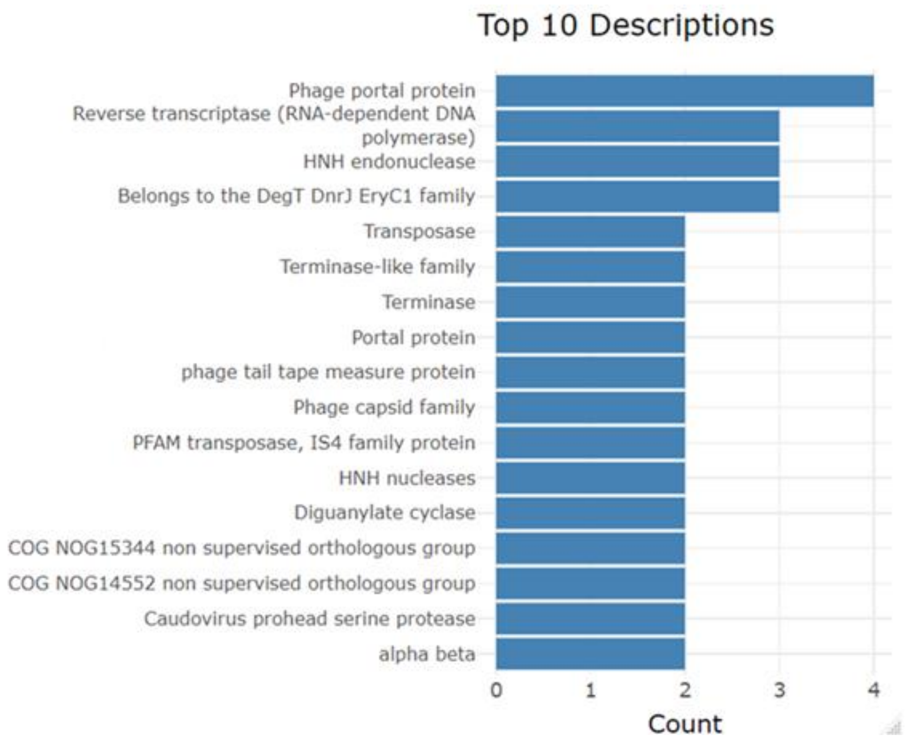

Figure S3: Distribution of the top 10 functional annotations of the metagenome in Standerton analysed using the EggNOG Dataset.
