# Supplementary material for: Elucidating the Functional and Taxonomic Diversity of Soil Microbial Communities From Three Commercial Soybean Farms in South Africa
Source: Environ Microbiol Rep. 2026 May 15;18(3):e70360. doi: 10.1111/1758-2229.70360 (PMC13178142; doi:10.1111/1758-2229.70360)
Supplement: Supplementary file 2 — Figure S4: comparison of the relative abundance of the top class (A) and family (B) of the soybean rhizosphere microbial communities between all the three locations. [file EMI4-18-e70360-s001.pdf]

A

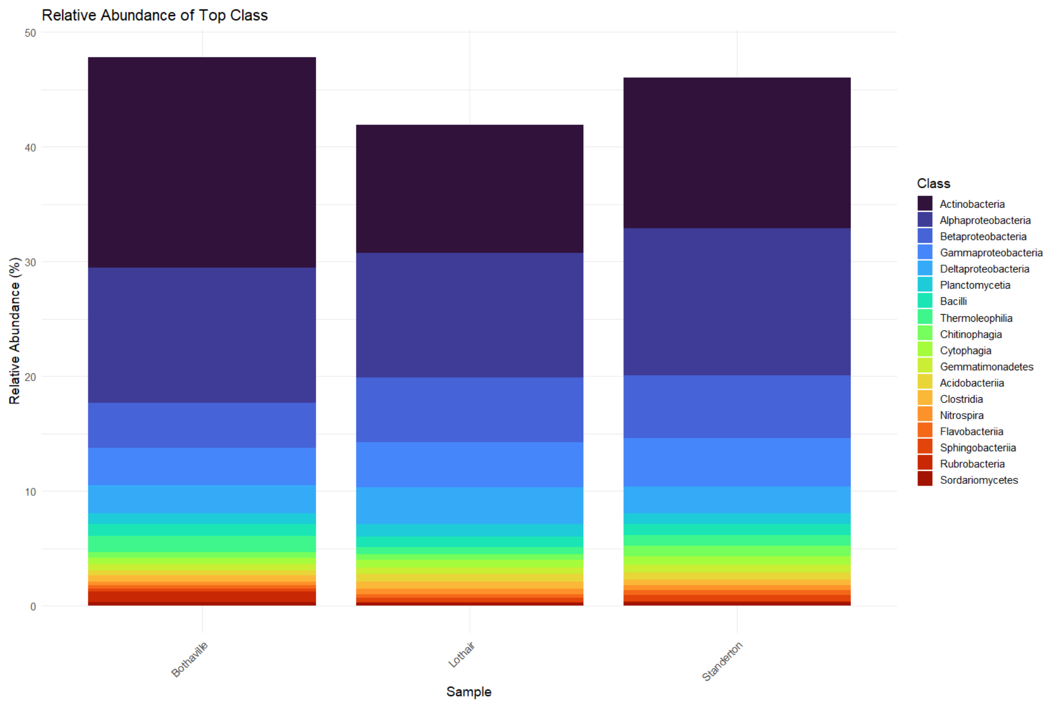

B

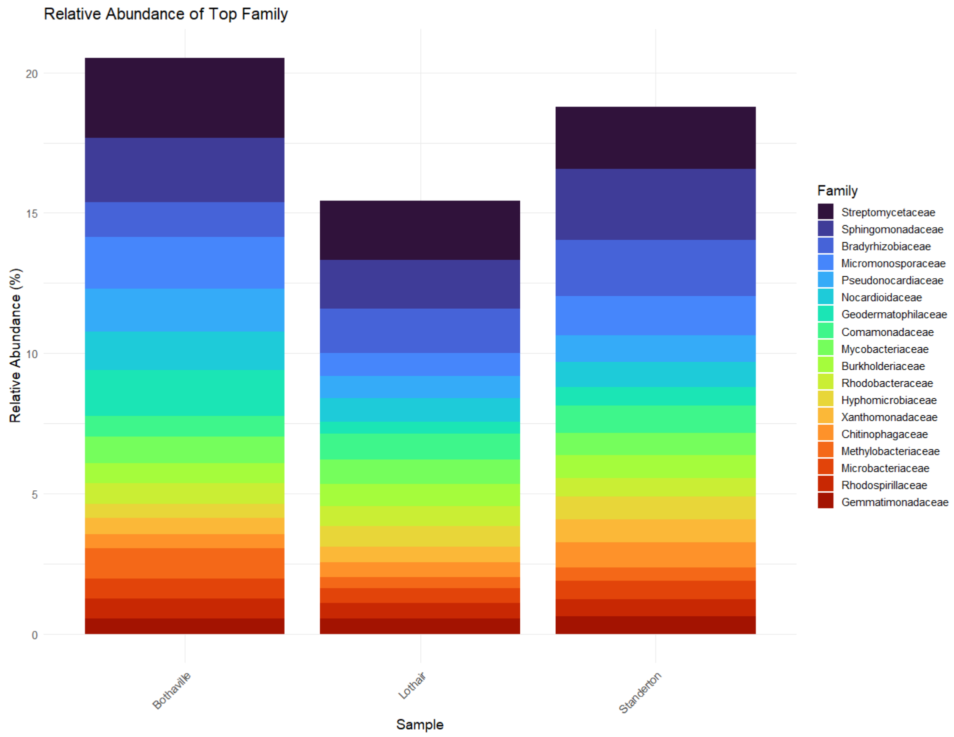

Figure S4: comparison of the relative abundance of the top class (A) and family (B) of the soybean rhizosphere microbial communities between all the three locations.
