# Supplementary material for: Elucidating the Functional and Taxonomic Diversity of Soil Microbial Communities From Three Commercial Soybean Farms in South Africa
Source: Environ Microbiol Rep. 2026 May 15;18(3):e70360. doi: 10.1111/1758-2229.70360 (PMC13178142; doi:10.1111/1758-2229.70360)
Supplement: Supplementary file 3 — Table S1: Physicochemical properties of the soybean rhizosphere soils from three locations. Table S2: Genome Assembly Metrics for Bothaville, Lothair and Standerton. [file EMI4-18-e70360-s003.pdf]

Supplementary Tables.

Table S1. Physicochemical properties of the soybean rhizosphere soils from three locations

**Farm Location**	**Standerton**		**Bothaville**		**Lothair**	
**Chemical analysis**	*Sample 1*	*Sample 2*	*Sample 3*	*Sample 4*	*Sample 5*	*Sample 6*
Organic C (%)	0.99	1.21	0.27	0.37	0.85	1.48
P (mg/kg)	40.11	49.96	46.34	45.40	33.41	45.43
Al (N/A)	0.011	0.010	0.018	0.014	0	0
Mn	60.4	61.2	20.7	31.8	40.5	43.3
N-No3	15.03	19.88	23.28	15.99	12.47	11.03
**Extractable cations**	**Standerton**		**Bothaville**		**Lothair**	
Na	0.020	0.019	0.010	0.010	0.015	0.018
K	1.266	1.279	0.509	0.844	0.427	0.655
Ca	6.737	7.735	1.477	2.570	7.934	4.925
Mg	1.234	1.917	0.650	0.897	1.283	1.193
S value	9.256	10.94	2.645	4.321	9.660	6.791
CEC	16.278	17.61	7.763	7.527	6.422	6.957
**Textual size**	**Standerton**		**Bothaville**		**Lothair**	
Sand	58.0	56.0	96.0	86.0	80.0	78.0
Silt	16.0	18.0	0.0	0.0	8.0	6.0
Clay	26.0	26.0	8.0	14.0	12.0	16.0
Soil pH	6.54	6.43	6.28	6.43	7.31	6.94

Table S2. Genome Assembly Metrics for Bothaville, Lothair and Standerton

Assembly Metric	Bothaville soil	Lothair soil	Standerton soil
# contigs (≥ 0 bp)	102096	71767	93283
# contigs (≥ 1000 bp)	308	105	213
# contigs (≥ 5000 bp)	1	4	4
# contigs (≥ 10000 bp)	1	2	2
# contigs (≥ 25000 bp)	1	0	0
# contigs (≥ 50000 bp)	0	0	0
Total length (≥ 0 bp)	32,115,918 bp	22,341,003 bp	29,433,696 bp
Total length (≥ 1000 bp)	444,942 bp	185,564 bp	342,999 bp
Total length (≥ 5000 bp)	43,561 bp	39,924 bp	41,948 bp
Total length (≥ 10000 bp)	43,561 bp	26,882 bp	28,145 bp
Total length (≥ 25000 bp)	43,561 bp	0	0
Total length (≥ 50000 bp)	0	0	0
# contigs (final)	3860	2066	3040
Largest contig	43,561 bp	16,624 bp	17,556 bp
Final total length	2,670,017 bp	1,371,560 bp	2,095,656 bp
GC content (%)	63.71%	62.81%	56.22%
N50	646 bp	607 bp	639 bp
N90	521 bp	516 bp	518 bp
auN	1462.9	1054.8	1017.7
L50	1473	807	1153
L90	3337	1796	2629
# N's per 100 kbp	42.81	102.07	45.67
